# Liver Function Test Abnormalities in Depressed Patients Treated with Antidepressants: A Real-World Systematic Observational Study in Psychiatric Settings

**DOI:** 10.1371/journal.pone.0155234

**Published:** 2016-05-12

**Authors:** Cosmin Sebastian Voican, Severine Martin, Céline Verstuyft, Emmanuelle Corruble, Gabriel Perlemuter, Romain Colle

**Affiliations:** 1 Faculté de Médecine Paris-Sud, Univ Paris-Sud, Université Paris-Saclay, Le Kremlin-Bicêtre, France; 2 INSERM U996, DHU Hepatinov, Labex LERMIT, Clamart, France; 3 Service d’hépato-gastroentérologie, Hôpital Antoine-Béclère, Hôpitaux Universitaires Paris-Sud, Assistance Publique-Hôpitaux de Paris, Clamart, France; 4 Equipe «Depression et Antidépresseurs», INSERM UMR-1178, Le Kremlin Bicêtre, France; 5 Service de Psychiatrie, Hôpital de Bicêtre, Hôpitaux Universitaires Paris-Sud, Assistance Publique-Hôpitaux de Paris, Le Kremlin Bicêtre, France; 6 INSERM U1184, Le Kremlin Bicêtre, France; 7 Service de Génétique moléculaire, Pharmacogénétique et Hormonologie, Hôpital de Bicêtre, Hôpitaux Universitaires Paris-Sud, Assistance Publique-Hôpitaux de Paris, Le Kremlin Bicêtre, France; The Chinese University of Hong Kong, HONG KONG

## Abstract

**Background:**

Concerning the risk of antidepressant induced liver injury, it is not clear whether psychiatrists perform a liver function test (LFT) and whether an increase in aminotransferase levels should contraindicate antidepressant treatment.

**Aim:**

To evaluate LFT availability, the prevalence of LFT abnormalities and the probable cause of an altered LFT in patients with a major depressive episode (MDE) requiring an antidepressant drug.

**Methods:**

We studied LFT evaluation in a real world psychiatric setting, in a sample of 321 consecutive patients with a current major depressive episode (MDE) requiring an antidepressant drug treatment, but without current alcohol or drug dependence or unstable medical disease.

**Results:**

An LFT is performed in 36.1% (116/321) of depressed patients. One fifth of antidepressant-treated patients who had an LFT evaluation had abnormal results. The most frequent causes of LFT abnormalities were: NAFLD (nonalcoholic fatty liver disease) (7/321; 2.1%), acute alcohol consumption (4/321; 1.2%), antidepressant-induced liver injury (3/321; 0.9%), hepatitis C virus infection (2/321; 0.6%) and heart failure (1/321; 0.3%). The cause of LFT abnormalities was unknown in 32% of patients (8/25) due to the absence of etiological investigations.

**Conclusion:**

These results demonstrate that an LFT is infrequently performed by psychiatrists in depressed patients requiring an antidepressant drug. Baseline LFT assessment and observations during the first six months of antidepressant treatment may be useful for detection of patients with pre-existing liver disease such as NAFLD, and early identification of cases of antidepressant-induced liver injury. An increase in aminotransferase levels may be related to an underlying liver disease, but does not contraindicate antidepressant treatment.

## Introduction

Drug-induced liver injury (DILI) is the fourth leading cause of liver damage in western industrialized countries (1.28–29 cases per 100,000 patient-years) and a matter of concern in the current context of increasing drug availability and medical prescription [1]. No specific markers are available and DILI is a diagnosis of exclusion. The first accepted sign for a DILI diagnosis is an increase of ALT (alanine aminotransferase) and AP (alkaline phosphatase) values temporally associated with the administration of the drug. In order to avoid unnecessary drug withdrawal, ALT>5x upper limit of normal (ULN) and/or AP>2xULN have been proposed as threshold values indicating a potential DILI [2, 3]. Once DILI is suspected, other causes of liver injury have to be excluded and several clinical scores have been developed to assess drug imputability [4–7].

Antidepressant-induced liver injury is a rare event but may be severe and irreversible[8]. Liver function is assessed in some clinical trials evaluating newer antidepressant (AD) agents. But patients included in clinical trials do not reflect patients treated with AD in real life settings. Therefore, the incidence of abnormal liver function tests (LFTs) or DILI during AD treatment in real life settings is difficult to estimate [8]. In clinical practice, an LFT is not routinely performed before or during AD treatment and recommendations exist only for recently commercialized AD such as agomelatine [9].

Asymptomatic mild abnormal LFTs are detected in 0.5–3% of patients with major depressive disorder (MDD) treated with AD [8]. The nonalcoholic fatty liver disease (NAFLD) associated with metabolic syndrome is a leading cause of transaminase increase and liver injury [10]. Several studies have shown an association between major depression and metabolic syndrome [11, 12, 13]. NAFLD may therefore be a cause of LFT abnormalities in patients treated with AD, but there are no studies addressing this issue. Patients with alcohol abuse or chronic viral hepatitis may also have increased ALT levels and need antidepressants. Therefore, an increase of transaminase levels in MDD may not be related to AD themselves in a DILI process, but to several other causes, which are not mutually exclusive. Nevertheless, studies evaluating the reasons for LFT abnormalities in MDD patients are insufficient.

In this observational study of depressed patients, we aimed to evaluate LFT availability, the prevalence of LFT abnormalities and the most probable cause of an altered LFT with a particular focus on DILI due to AD treatment in MDD.

## Patients and Methods

### Patients

We retrospectively assessed the clinical records of 321 consecutive patients (90% Caucasians); however, they were initially prospectively included in the METADAP cohort study between 2011 and 2014 [13]. The METADAP study aimed to assess the incidence of metabolic syndrome in patients with Major Depressive Episode (MDE) treated with antidepressants. Patients were eligible for inclusion if they were between 18 and 65 years-old, had a current MDE in a context of MDD [Diagnostic and Statistical Manual of Mental Disorders, Fourth Edition Text Revision (DSM-IVTR) diagnostic criteria] with a Hamilton Depression Rating Scale (HDRS) score > 18, requiring an index prescription of AD. They were admitted to the hospital in the psychiatry department or followed up by a psychiatrist in the hospital outpatient service. The exclusion criteria were bipolar disorder, psychotic disorder, eating disorders (DSM-IVTR diagnostic criteria), psychiatric symptoms of a somatic disorder, unstable somatic disorder, pregnancy, treatment with a mood stabilizer, long-term antipsychotic treatment (prescribed for more than 4 months during the last year), legal protection, current alcohol (daily alcohol consumption ≥30 g for men and ≥20 g for women) or drug dependence, and participation in another research protocol in the last two months. Written informed consent was obtained from all participants. The study was conducted in accordance with the French law concerning medical investigations (Huriet Law) and the Helsinki declaration. The protocol was approved by the ethics committee of Bicêtre Hospital.

### Antidepressant treatment

Patients were initiated with a monotherapy of AD treatment, all marketed AD on France being considered. The AD treatment included a drug from one of the four AD drug classes: selective serotonin reuptake inhibitor(s) (SSRI) (citalopram, escitalopram, paroxetine, fluoxetine, sertraline), serotonin-norepinephrine reuptake inhibitor(s) (SNRI) (venlafaxine, duloxetine, minalcipran), imipraminic medications (clomipramine, amitriptyline, maprotiline), or other AD medications (mianserine, mirtazapine, agomelatine). Other medications were allowed except mood stabilizers and antipsychotics. None of the hospitalised patients were admitted for suicide/self-harm attempt by drug overdose.

### Definition of LFT abnormalities and DILI

The METADAP cohort was not intended to assess liver function and the decision to perform an LFT was not influenced by inclusion in the study.

In most cases, DILI related to AD use occurs during the first six months of treatment [8]. Therefore, we retrospectively collected the LFT [ALT, AST (aspartate aminotransferase), AP, gamma-glutamyl transferase and bilirubin] available at baseline (includes the six months before study inclusion) and during the first six months of AD treatment. Abnormal LFTs were defined by the presence of at least one of the following variables above the ULN provided by the local laboratory: ALT>45 IU/l, AST>40 IU/l, AP>130 IU/l and total bilirubin > 17 μmol/l. For patients with an abnormal LFT, the available etiologic data were collected: alcohol consumption (quantified as self-reported drinking habits; patients' families were also interviewed, when possible; no blood markers for alcohol consumption such as carbohydrate-deficient transferrin were performed), presence of NAFLD, viral hepatitis [immunoglobulin M antibodies (IgM) to hepatitis A, IgM to hepatitis B core antigen, antibodies to hepatitis C and hepatitis C viral load, IgM to hepatitis E and hepatitis E viral load, IgM to cytomegalovirus, IgM to Epstein-Barr virus, IgM to herpes viruses], ongoing medications, hemochromatosis (serum ferritin level and transferrin saturation), Wilson disease (serum ceruloplasmin level and urinary copper excretion), autoimmune hepatitis (diagnosis based on previously validated criteria)[14], bile duct obstruction (abdominal ultrasound), liver ischemia (clinical context and transthoracic echocardiography), hepatic arterial or venous obstruction (abdominal ultrasound examination with Doppler). Patients without available etiologic exploration were classified as LFT abnormalities of unknown etiology. Alcohol consumption was evaluated at inclusion as a daily alcohol intake ≥30 g for men and ≥20 g for women was one of the exclusion criteria. A new evaluation of daily alcohol consumption was done when LFT abnormalities were detected. Acute alcohol consumption was defined as a relapse from alcohol during the study period. The diagnosis of NAFLD was based on the following criteria: evidence of steatosis on liver ultrasound, daily alcohol consumption <30 g for men and <20 g for women, abnormal LFT, presence of metabolic syndrome components and no other cause of liver steatosis [15, 16]. DILI was suspected in patients with ALT values > 5xULN or AP values > 2xULN [3] which were temporally associated with the administration of the antidepressant drug. Drug imputability was assessed by Roussel Uclaf Causality Assessment Method (RUCAM) scale [4, 17], a widely used method to quantify the strength of association between liver injury and implicated medication. The RUCAM scale is composed of several different criteria including: temporal relationship between the drug intake and development of an abnormal LFT, evolution of LFTs following drug withdrawal, alcohol consumption, age, previous case reports of DILI, concomitant medication, exclusion of all potential causes of liver damage, drug rechallenge. A semi-quantitative score was calculated and an association between AD drug use and liver injury was defined as follows: highly probable (score>8); probable (score = 6–8); possible (score = 3–5); unlikely (score = 1–2); excluded (score<0). Data collection was carried out independently by two investigators (CSV and SM) under the supervision of GP and EC. The investigators were blind from the results of the METADAP cohort.

### Definition of metabolic syndrome

Metabolic syndrome was evaluated using clinical and biologic criteria: waist circumference, blood pressure, fasting blood glucose, lipid profile, use of antihypertensive, lipid-lowering medications or glucose lowering drugs. We used the definition from the National Cholesterol Education Program, 3 of the following criteria had to be met: waist circumference >102 cm in men and >88 cm in women, triglycerides ≥1.7 mmol/l, HDL (high density lipoprotein) cholesterol <1.03 mmol/l in men and <1.29 mmol/l in women, systolic pressure ≥130 mmHg or diastolic pressure ≥85 mmHg, fasting plasma glucose ≥6.2 mmol/l) [18].

### Evaluation of CYP450 and glutathione S-transferase polymorphisms

For patients with an available LFT, we assessed the polymorphisms of main cytochrome P450 (CYP) and glutathione S-transferase (GST) isoenzyme involved in AD metabolism. Genomic DNA was extracted from circulating leukocytes using the Puregene Kit (Gentra systems, MN, USA). CYP2D6 polymorphisms referred to the CYP Allele Nomenclature Committee (http://www.cypalleles.ki.se). CYP2D6 pharmacogenetic analysis used defined genotype-phenotype relationships based on known biochemical and pharmacological effects and included major CYP2D6 alleles within a population of European descendants [19]. We referred to the following phenotypes: poor metabolizers (PM)—lacking active enzyme function, homozygous or compound heterozygous for CYP2D6*3, *4, or *5alleles; intermediate metabolizers (IM)—reduced enzyme activity, carrying *10 and *41 alleles either homozygous or in combination with a PM allele. Patients were genotyped for the genetic polymorphisms: CYP2D6*3 (rs35742686), CYP2D6*4 (rs3892097), CYP2D6*6 (rs5030655), CYP2D6*10 (rs1065852), CYP2D6*41 (rs28371725), CYP2C19*2 (rs4244285), CYP2C19*3 (rs4986893), CYP2C19*17 (rs12248560), CYP3A4*22 (rs35599367), CYP3A5*3 (rs776746), CYP1A2*1F (rs762551), blind from clinical data. The described genetic polymorphisms referred to the international list described by the CYP Allele Nomenclature Committee (http://www.cypalleles.ki.se). Single nucleotide polymorphisms (SNPs) detection and deletion were performed using Taqman® allelic discrimination assay (ABI prism 7900HT, Applied Biosystem, Courtaboeuf, France), as previously described [20]. The CYP2D6 duplication and deletion (CYP2D6*5) were determined using the real time PCR TaqMAn 7900HT Applied Biosystems Instrument [21]. GSTT1, GSTP1, GSTM1 genotyping was performed by genomic-level polymerase chain reaction (PCR) with restriction fragment length polymorphism analysis. All patients were treated in duplicate. The informed consent for DNA tests was obtained from all patients. Approval by ethics committee of Bicêtre Hospital was also obtained.

### Statistical analysis

Quantitative variables are expressed as mean and standard deviation. Qualitative variables are expressed as percentages. Chi-squared tests were used to compare qualitative variables. Student’s t-tests were used to compare normally distributed quantitative variables and the Mann–Whitney test was used to compare quantitative variables that were not normally distributed. GraphPad Prism software was used for statistical analyses.

## Results

### Patients who were prescribed a LFT

In this real life cohort of 321 patients with a MDE treated by psychiatrists, LFTs were not systematically performed in patients treated with AD drugs. Overall, 116 patients (36.1%) had at least one LFT before or during the first 6 months of treatment. Baseline LFTs were available in 84.4% (98/116) of patients with LFT evaluation while 16.6% (18/116) had a LFT evaluation only after AD treatment initiation. At least two LFT determinations were available in 9% of patients (29/321). Table 1 summarizes the differences between patients who benefited or not from an LFT as prescribed by their psychiatrist. As compared to those who did not, those who benefited from an LFT evaluation before or during AD treatment were more likely to be inpatients and had significantly higher serum triglyceride levels. The frequency of metabolic syndrome was also higher, but the difference was not statistically significant. However, they did not differ in terms of age, gender, history of MDD and previous AD treatments, current MDE severity as rated by the HDRS score, and current AD treatment.

**Table 1:** Characteristics of patients who were prescribed a LFT by their psychiatrist.

	*All (n = 321)*	*LFT prescribed (n = 116)*	*No LFT prescribed (n = 205)*	*p*
**Age, years (SD)**	46 (12.8)	46.7 (12.7)	45.6 (12.9)	NS
**Sex (M/F)**	99/222	37/79	62/143	NS
**Caucasians**	90%	93%	90.2%	NS
Hospitalized *	82.5%	89.5%	78.6%	0.01
**Recurrent MDD**	73.8%	76.5%	73.3%	NS
**Age at onset of MDD, years (SD)**	33.3 (13.8)	32.9 (12.8)	33.5 (14.4)	NS
**History of AD use**	81.9%	83.4%	81%	NS
**HDRS (SD)**	23.9 (4.6)	24 (4.2)	23.8 (4.9)	NS
**QIDSC (SD)**	22.4 (5.5)	22.9 (5.2)	22.2 (5.7)	NS
**QIDSSR (SD)**	21.3 (7)	21.6 (6.7)	21.2 (7.2)	NS
**SSRI**	37.4%	38.3%	36.8%	NS
**SNRI**	43.1%	43.7%	42.7%	NS
**IMI**	11%	10.7%	11.2%	NS
**Other antidepressants**	8.5%	7.3%	9.3%	NS
**Tobacco**	36.4%	40.8%	33.9%	NS
**BMI, kg/m**^**2**^ **(SD)**	24.2 (4.7)	24.4 (4.5)	24 (4.9)	NS
**WC, cm (SD)**	90.7 (13.7)	92.2 (12.4)	89.8 (14.3)	NS
**Dyslipidemia**	19%	19.8%	18.5%	NS
**Hypertension**	21.8%	23.2%	20.9%	NS
**Diabetes**	4.3%	4.3%	4.3%	NS
**Triglycerides, mmol/l (SD)**	1.1 (0.7)	1.3 (0.9)	1.1 (0.5)	<0.01
**LDL cholesterol, mmol/l (SD)**	1.2 (0.3)	1.2 (0.4)	1.2 (0.3)	NS
**HDL cholesterol, mmol/l (SD)**	0.5 (0.1)	0.5 (0.1)	0.5 (0.1)	NS
**FPG, mmol/l (SD)**	0.9 (0.2)	0.9 (0.1)	0.9 (0.2)	NS
**Metabolic syndrome**	24.5%	27.8%	22.7%	NS

*Abbreviations*: LFT, liver function tests; SD, standard deviation; NS, non significant; AD, antidepressant; MDD, major depressive disorder; HDRS, Hamilton depression rating scale; QIDSC, quick inventory of depressive symptomatology clinician rated; QIDSSR, quick inventory of depressive symptomatology self-report; SNRI, Serotonin–norepinephrine reuptake inhibitors; SSRI, Selective serotonin re-uptake inhibitors; IMI, imipraminic antidepressants; BMI, body mass index; WC, waist circumference; LDL, low density lipoprotein; HDL, high density lipoprotein; FPG, fasting plasma glucose.

### Prevalence of LFT abnormalities in patients who benefited from an LFT

LFT abnormalities were found in 21.5% of patients with available LFTs (25/116). Thus the frequency of LFT abnormalities is at least 7.8% (25/321) in this sample of MDD patients with a current MDE treated in psychiatric settings. In most cases (18/25 patients, 72%), LFT abnormalities were detected before AD treatment initiation, thus excluding antidepressant-induced injury.

### Etiology of LFT abnormalities

We evaluated the available etiological data in order to establish the cause of LFT abnormalities. NAFLD was the most probable cause in 28% of cases (7/25). Other causes of LFT abnormalities were: acute alcohol consumption 16% (4/25), hepatitis C virus infection 8% (2/25), and heart failure 4% (1/25). The four patients with LFT abnormalities attributed to alcohol consumption, relapsed from alcohol (>50 g/day) during the study and no other cause of liver enzyme elevation was found. In patients with LFT abnormalities attributed to heart failure, transthoracic echocardiography showed global ventricular failure and other causes of mild liver enzyme elevations (2.5xupper limit of normal) were excluded. LFT improved with treatment of heart failure. Of note, etiological investigations were not carried out in one third of patients (8/25), all of these patients having mild LFT abnormalities (<3xULN). None of these patients had liver biopsy examination.

The prevalence of NAFLD with an abnormal LFT was 2.1% (7/321) in the overall cohort and 6% (7/116) in patients who were prescribed an LFT. The prevalence of AD-induced liver injury was at least 0.9% (3/321) in the overall cohort. In patients who were prescribed an LFT (n = 116), the prevalence of AD-induced liver injury was 2.5% (3/116). DILI represented 12% (3/25) of all causes of abnormal LFTs.

Since NAFLD is strongly associated with metabolic syndrome, we assessed the differences in terms of metabolic syndrome and metabolic syndrome components (Table 2). We showed that the prevalence of metabolic syndrome was significantly higher in antidepressant-treated patients with an abnormal LFT than in those with a normal LFT (44% vs. 23.3%, p = 0.04), with higher triglycerides serum levels (p = 0.03) (Table 2).

**Table 2:** Characteristics of patients with normal and abnormal LFT.

	*Abnormal LFT (n = 25)*	*Normal LFT (n = 91)*	*p*
**Age, years (SD)**	48.4 (10.9)	46.3 (13.2)	NS
**Sex (M/F)**	11/14	26/65	NS
**BMI, kg/m**^**2**^ **(SD)**	25 (4.3)	24.3 (4.5)	NS
**WC, cm (SD)**	95.7 (11.9)	91.2 (12.5)	0.06
**Dyslipidemia**	16%	20.8%	NS
**Hypertension**	36%	19.7%	0.08
**Diabetes**	8%	3.2%	NS
**Triglycerides, mmol/l (SD)**	1.6 (1.2)	1.2 (0.8)	0.03
**Total cholesterol, mmol/l (SD)**	2 (0.4)	1.9 (0.4)	NS
**HDL, mmol/l (SD)**	0.5 (0.1)	0.5 (0.1)	NS
**FPG, mmol/l (SD)**	0.9 (0.2)	0.9 (0.1)	NS
**Metabolic syndrome**	44%	23.3%	0.04

*Abbreviations*: LFT, liver function tests; BMI, body mass index; WC, waist circumference; HDL, high density lipoprotein; FPG, fasting plasma glucose; NS, non significant.

The diagnosis of DILI due to antidepressant drug was suspected in 3 patients with an increase of ALT>5xULN upon initiating antidepressant drug treatment. The three cases of probable DILI are presented in Tables 3 and 4.

**Table 3:** Description of the 3 cases of antidepressant-induced liver injury.

	Case 1 (55-year-old woman)	Case 2 (40-year-old man)	Case 3 (47-year-old man)
**Antidepressant**	Escitalopram	Venlafaxine	Amitriptyline
**Daily dose**	10 mg	150 mg	50 mg
**Concomitant medication**	Chlorpromazine	Zopiclone	Cyamemazine, Alimemazine
**Type of lesion**	Hepatocellular	Mixed	Mixed
**Latency**	4 days	11 days	10 weeks
**Clinical context**	Flu-like syndrome, hypereosinophilia	Asymptomatic	Asymptomatic
**Medical history**	Unremarkable	Sleep disorders	Unremarkable
**Antidepressant withdrawal**	Yes	Yes	Yes
**Outcome**	Complete recovery	Complete recovery	Complete recovery
**RUCAM causality score**	8 (probable)	7 (probable)	5 (possible)
**CYP450 polymorphism**	GST M1 deletion (loss of function)	Homozygous: CYP2D6*10 allele (reduced enzymatic activity)GST M1 deletion (loss of function)	Heterozygous: CYP2D6*4 (inactivating allele); CYP2C19*17 (ultra fast metabolizer allele)Homozygous: CYP1A2*1C (slow metabolizer allele)

*Abbreviations*: RUCAM, Roussel Uclaf Causality Assessment Method; CYP, cytochrome P450.

**Table 4:** Evolution of liver function tests in the 3 cases of antidepressant-induced liver injury.

	Case 1 (escitalopram-induced liver injury)				Case 2 (venlafaxine-induced liver injury)			Case 3 (amitriptyline-induced liver injury)			
	Index	Day 8	Day 12	Day 31	Index	Day 20	Day 59	Index	D37	Day 72	Day 133
**ALT (IU/l)**	17	797	319	36	46	232	147	39	320	100	55
**AST (IU/l)**	19	804	71	25	25	129	59	26	105	33	34
**ALP (IU/l)**	77	143	235	155	71	180	91	56	221	65	56
**Total bilirubin (μmol/l)**	17	19	13	9			5	17	7		
**INR**	1.1	1			1	1		1.1	1	1	

Abbreviations: ALT, alanine aminotransferase; AST, aspartate aminotransferase; ALP, alkaline phosphatase; INR, international normalized ratio.

### CYP450 and glutathione S-transferase polymorphisms in antidepressant treated patients

CYP2D6 genotype frequencies in the group of patients with an available LFT were as follows: CYP2D6*3–0% homozygous, 2.6% heterozygous; CYP2D6*4–0% homozygous, 28.4% heterozygous; CYP2D6*5–7.8% duplication or deletion; CYP2D6*6–0% homozygous, 1.7% heterozygous; CYP2D6*10–7.8% homozygous, 23.3% heterozygous; CYP2D6*41–0% homozygous, 14.7% heterozygous. CYP2D6*10 allele was present in a homozygous state in the patient with venlafaxine-related DILI. CYP2D6*10 homozygosity was found in 33.3% (1/3) of patients with antidepressant-induced liver injury, but only in 7.8% (9/116) of patients with an available LFT. CYP2D6*4 and CYP2C19*17 were present in a heterozygous state in the patient with amitriptyline-related DILI. This genotype association was found in 33.3% (1/3) of patients with DILI, but only in 9.5% (11/116) of patients with an available LFT. GST M1 deletion was present in 2 of the 3 patients with antidepressant-related DILI. However, the frequency of this deletion was similar in DILI patients and patients with available LFT (66.6% and 52%, respectively).

## Discussion

In this observational cohort study, we showed that evaluation of an LFT is infrequently performed in depressed patients without current alcohol dependence, drug abuse or unstable medical conditions, which require starting an antidepressant treatment, by a psychiatrist. This suggests that psychiatrists are not aware of antidepressant induced DILI. We were not able to identify clear criteria for assessment of LFTs except that they were more often conducted with inpatients, and had higher triglyceride serum levels. Nevertheless, the prevalence of LFT abnormalities was high reaching at least 7.8% of the overall cohort. Recommendations are therefore needed for assessment of LFT in depressed patients treated with antidepressants beyond those with current alcohol dependence, drug abuse or unstable medical conditions.

There are no recommendations regarding liver function testing in patients taking antidepressant medication. Furthermore, clinical trials evaluating LFTs exist only for newer drugs. Therefore, DILI related to antidepressant use is probably underdiagnosed in clinical practice [8]. In our cohort, only 36.1% of antidepressant-treated patients had at least one liver function testing available at baseline and/or during the first 6 months of treatment. Interestingly, 21.5% of these patients had LFT abnormalities. Most patients showed an altered LFT at baseline arguing against a drug-induced hepatic toxicity. Metabolic syndrome and metabolic syndrome components (waist circumference, hypertension and serum triglycerides) were more frequent in antidepressant-treated patients with LFT abnormalities. Moreover, NAFLD was the major cause of abnormal LFT in this group of patients. Acute alcohol consumption and chronic hepatitis C virus infection were also involved. Alcohol dependence is present in up 20% of patients with depression and may negatively affect the course of depressive disorders [22]. The METADAP cohort included patients without ongoing alcohol consumption. Nevertheless, alcohol relapse was the second most common cause of LFT abnormalities and should be considered in antidepressant-treated patients. These data suggest that an increase in aminotransferase levels (before any antidepressant treatment or during treatment) may be related to an underlying liver disease, not necessarily severe, that does not contraindicate antidepressant treatment. Therefore, assessment of baseline LFTs may be useful as they help to interpret the abnormal LFT results during antidepressant treatment. Abnormal results may be a manifestation of either underlying liver disease or antidepressant-induced hepatotoxicity.

In 32% of cases, LFT abnormalities were of unknown etiology. These patients did not meet the criteria for metabolic syndrome. Moreover, etiological data such as viral hepatitis status, autoimmune hepatitis markers, ultrasound exploration or liver biopsy were not available. The accepted thresholds for initiating causality evaluation in suspected DILI are ALT/AST > 5XULN, ALP > 2XULN or bilirubin > 2XULN [3]. The mild LFT abnormalities (<3xULN) in this group of patients may therefore explain the dearth of etiological evaluations. On the other hand, mild elevations in levels of the liver enzymes (<3xULN) are found in 1–5% of the general population [23]. Furthermore, physiological variations of aminotransferase (between 1xULN to 3xULN) levels can occur in up to 20% of normal subjects treated with placebo and followed up for a period of 2 weeks [24]. An increase of carbohydrate or fat intake may also lead to an increase of baseline aminotransferase levels in only three days [25]. In our cohort, patients with an abnormal LFT of unknown etiology had no further LFT surveillance. For this reason, we cannot exclude that these LFT abnormalities were related to physiological variations, the concomitant medication or the previous exposure to antidepressants. Surveillance of the LFT once abnormalities are detected may therefore be useful in patients receiving antidepressant medication to discriminate between physiological variations and possible drug-related toxicity.

A French prospective community study showed a global crude annual incidence of DILI of 13.9 cases per 100,000 inhabitants, 16 times higher than spontaneous case reporting rate to national regulatory authorities [26]. A more recent study conducted in medical inpatients reported a DILI incidence of 1.4% [27]. Carvajal Garcia-Pando and colleagues [28] reported an incidence of DILI associated with antidepressants requiring hospitalization of 1,28–29 cases per 100,000 patient-years. This estimation was based on cases of hepatic damage collected via spontaneous reporting and included in the Spanish Pharmacovigilance database, suggesting a possible underestimation of the disease. In our study, we report one case of symptomatic DILI associated to escitalopram and two cases of asymptomatic LFT abnormalities which fulfilled the criteria of DILI attributable to venlafaxine and amitriptyline use. The three patients developed increased ALT serum levels > 5xULN following antidepressant initiation that normalized upon treatment withdrawal and liver biopsy was unnecessary. LFT normalized despite continuation of concomitant medication (Table 3). However, drug-drug interactions involving CYP450 pathway cannot be excluded in these cases of DILI. In our cohort of depressed patients, the frequency of DILI associated with antidepressant use was 0.9% during the first six months of treatment. Considering that LFT were assessed in a minority of antidepressant-treated patients, undiagnosed cases of asymptomatic DILI may exist. Treatment continuation despite hepatotoxicity development could lead to severe hepatic failure or chronic hepatocellular dysfunction [8, 29–31]. All these data suggest that DILI related to antidepressant use is probably underestimated and surveillance of LFT in the first six months of treatment could improve detection of asymptomatic cases. However, routine LFT testing in antidepressant-treated patients may have major cost implications. Our study was not intended to evaluate the cost implications of performing LFT in depressed patients starting antidepressant treatment. Further studies are necessary to evaluate cost-effectiveness of LFT surveillance.

Generation of toxic metabolites is considered one of the mechanisms of drug-related hepatotoxicity. The metabolic pathway of antidepressant drugs includes CYP450 enzyme complex and GST. Genetic polymorphisms of CYP450 and GST isoenzymes including SNPs, duplications, deletions and gene conversions can cause either increased or reduced enzymatic activity levels [32, 33] with potential implication in DILI pathogenesis. Amitriptyline is demethylated mainly by CYP2C19 and CYP3A4 to form the active metabolite nortriptyline which is further metabolized by hydroxylation through the CYP2D6 pathway [34]. It was shown that nortriptyline but not amitriptyline serum levels correlated with adverse events [35]. Furthermore, increased CYP2C19 activity in combination with diminished CYP2D6 enzymatic activity was associated with high nortriptyline serum levels and high risk of adverse events [35], but the relationship with DILI is unknown for this drug. In this study, we showed that the patient who developed DILI associated with amitriptyline was heterozygous for ultra fast metabolizing allele CYP2C19*17 and inactivating allele CYP2D6*4. This genotype association was present in only in 9.5% of patients with an available LFT. Venlafaxine is metabolized primarily by CYP2D6, CYP3A4 and CYP2C19 [34]. Pharmacokinetics of venlafaxine is affected by CYP2D6*10 polymorphism which is a poor metabolizing allele [36]. The CYP2D6*10 allele was found in a homozygous status in a patient with DILI related to venlafaxine use. The patient was a homozygous carrier for the deletion GST M1 which leads to complete absence of enzymatic activity and potentially higher susceptibility to toxic liver injury. CYP450 and GST polymorphisms may therefore play a role in the pathogenesis of hepatic toxicity related to antidepressant use, but further studies are needed.

Our study has several limitations. First, it is a retrospective review of a prospectively acquired database. Nevertheless, a primary goal was to assess frequency of LFT availability in depressed patients requiring antidepressants in psychiatric clinical practices. Second, the number of patients was relatively low to evaluate DILI associated with antidepressant drug use which is a rare condition.

In conclusion, this study demonstrates that LFTs are infrequently performed in depressed patients requiring AD and treated by psychiatrists. Baseline LFT assessments and follow-up during the first six months of treatment with AD may be useful for detection of patients with pre-existing liver disease such as NAFLD, and early identification of cases of antidepressant-induced liver injury. Asymptomatic cases of DILI associated with antidepressant use may be relatively frequent and much effort should be done to avoid irreversible liver damage. Our results also suggest that an increase in aminotransferase levels may be related to an underlying liver disease, not necessarily severe, but does not contraindicate antidepressant treatment. Recommendations are needed for assessment of LFTs in depressed patients treated with antidepressants.
